# Predicting ankylosing spondylitis disease activity via patient-reported outcome measures: Building prediction models based on machine learning

**DOI:** 10.1371/journal.pone.0353486

**Published:** 2026-07-15

**Authors:** Yifan Gong, Aomei Liu, Li Zhuo, Xueyuan Xu, Hongxiao Liu

**Affiliations:** 1 Guang’anmen Hospital, China Academy of Chinese Medical Sciences, Beijing, China; 2 The School of Information Science and Technology, Beijing University of Technology, Beijing, China; Vanderbilt University, UNITED STATES OF AMERICA

## Abstract

**Objective:**

Disease activity is a critical indicator for monitoring the progression of ankylosing spondylitis (AS), guiding clinical decision-making, and informing treatment plans. Patient-reported outcome measures (PROMs) have gained prominence in AS clinical management. However, their potential to predict Ankylosing Spondylitis Disease Activity Score-C-reactive protein (ASDAS-CRP) remains unexplored. This study employs machine learning (ML) techniques to develop prediction models utilizing PROMs data to estimate disease activity in patients with AS.

**Methods:**

We utilized data from 389 patients with AS were included sourced from the China Rheumatoid Arthritis Registry of Patients with Chinese Medicine (CERTAIN) from March 2022 to March 2024. This dataset was divided into a training set (80%) and a testing set (20%). A total of 34 variables, including clinician-recorded features and PROMs (e.g., BASDAI, BASFI, BASMI, PGA, VAS, ASAS-HI, FACIT-F, DASS-21), were employed for feature selection and assessment of feature significance using a variety of machine learning methods. Ten models were constructed using Support Vector Machine (SVM) and K-Nearest Neighbour (KNN) classifiers in conjunction with five feature selection methods: Feature Selection with Orthogonal Regression (FSOR), Trace Ratio Criterion (TRC), Robust Feature Selection (RFS), Pearson Correlation Coefficient (PCC), and ReliefF. Model performance was evaluated based on accuracy, specificity, sensitivity, and area under the receiver operating characteristic curve (AUC-ROC).

**Results:**

A total of 389 patients with AS were included in the analysis. Key characteristics assessed included Patient Global Assessment (PGA), age, and the impact of disease on daily activities. The results indicated that the FSOR+SVM model achieved the best overall performance, with an AUROC of 0.930 (95%CI: 0.87–0.99) in the validation set. Meanwhile, FSOR+SVM also exhibited the highest sensitivity (83.78%), accuracy (79.35%), and specificity (90.50%).

**Conclusion:**

The machine learning model developed from PROMs data proved effective for predicting AS disease activity, showing strong agreement with clinical ASDAS-CRP measures.

## 1. Introduction

Ankylosing Spondylitis (AS) is a chronic autoimmune disorder that causes progressive inflammation of the sacroiliac joints and spinal ankylosis. The AS has a global prevalence ranging from 0.1% to 0.5% [1]. Early symptoms typically include lower back pain and stiffness. Without timely intervention, disease progression can result in severe spinal deformities, significantly impacting patients’ quality of life and prognosis. Despite ongoing research, a definitive cure for AS has not yet been found. Early diagnosis and treatment are critical for controlling disease progression. Current treatments primarily aim to slow disease advancement and enhance patients’ quality of life.

Disease activity in AS is a crucial indicator for assessing disease progression and guiding therapeutic strategies. The 2016 Assessment of SpondyloArthritis International Society (ASAS) and European League Against Rheumatism (EULAR) Management Guidelines introduced a new treatment objective: achieving low or inactive disease activity in AS [2]. Disease activity plays a pivotal role in enabling clinicians to assess a patient’s condition and inform therapeutic decisions. Different levels of disease activity are associated with distinct clinical manifestations and disease states. Currently, disease activity assessment largely relies on clinical evaluations and laboratory tests, such as symptom scores and inflammatory markers, particularly C-reactive protein (CRP). The 2017 International ASAS Working Group recommended the Ankylosing Spondylitis Disease Activity Score (ASDAS), with ASDAS-CRP being the primary tool to assess disease status in AS [3]. The ASDAS-CRP score is derived from a weighted evaluation of several factors, including low back pain, external joint pain, nocturnal pain, and CRP levels. Despite its widespread use, the ASDAS-CRP method has several practical limitations. For example, some rheumatology outpatients with AS cannot obtain comprehensive laboratory data or are inconvenient to visit hospitals and cannot undergo laboratory tests, making the calculation of ASDAS-CRP difficult. This constraint can hinder accurate monitoring of disease progression, potentially affecting medical decisions. Therefore, there is a pressing need to develop alternative methods that can accurately and conveniently predict disease activity without relying on laboratory data.

Recently, PROMs have become increasingly important in clinical practice and research trials [4]. PROMs are self-reports of a patient’s health status that come directly from the patient and are not influenced by the clinician or others, making them free from clinician or third-party interpretation [5]. Patients are the most reliable sources for reporting their health experiences. These measures provide a direct and intuitive account of patients’ experiences with their disease or treatment, capturing subjective symptoms such as pain, fatigue, or anxiety—information that laboratory tests may not detect by laboratory tests [6]. PROMs are not only quicker and less burdensome than clinical assessments, but they are also more cost-effective.

PROMs provide valuable insights into a patient’s health status and quality of life and have been recognized as essential outcome indicators for many rheumatic diseases [7]. They were widely used in clinical practice [8]. A variety of self-report tools have been developed to measure disease-specific outcomes in AS. For example, physical function is often assessed using the Bath Ankylosing Spondylitis Functional Index (BASFI) and is often used as a key secondary outcome in clinical trials of AS treatments, as well as being recommended for inclusion in assessments in clinical practice [9]. Real-world studies have shown that PROMs have strong correlations with traditional measures of AS disease activity, such as ASDAS [10]. This suggests that PROMs may be able to complement or even replace traditional AS disease activity measures. Unlike traditional measures, which require patients to be physically present at the clinic and undergo laboratory tests, PROMs can be implemented through physician assessment of patients on a scale. This eliminates the need for laboratory data and allows for a more patient-centered approach. Therefore PROMs-based disease activity measures may help overcome the limitations of traditional measures and provide clinicians with a better understanding of the patient’s condition from their perspective.

Disease activity prediction models based on PROMs are emerging as an effective clinical tool with the widespread use of machine learning (ML) technology in detecting, stratifying, and predicting at-risk populations and disease progression [11, 12]. By utilizing ML approaches, personalized predictions based on PROMs and basic patient information can serve as a valuable alternative to traditional disease activity measures. This has the potential to support clinical decision-making and improve treatment response assessments. While some researchers have applied ML algorithms to assess rheumatic diseases, there is still a lack of systematic integration of data from multiple PROMs and routine physician records for comprehensive prediction of AS disease activity [13–16].

In this study, we developed an efficient machine-learning model that could accurately predict AS disease activity without relying on laboratory data. By integrating multiple PROMs with routinely recorded clinical features, this model offers a practical tool for clinicians to monitor patient conditions and make informed adjustments to treatment plans, ultimately improving patient outcomes. PROMs play a pivotal role in this process, providing crucial insights into patients’ subjective health experiences and significantly contributing to the accurate prediction of disease activity.

## 2 Objects and methods

### 2.1 Subjects

This study utilized AS data from the China Rheumatoid Arthritis Registry of Patients with Chinese Medicine (CERTAIN) collected between March 2022 and March 2024. The data were accessed for research purposes on 20/05/2024. A total of 389 AS outpatients and inpatients were studied across 10 hospitals in seven regions, including Guang’anmen Hospital, China Academy of Traditional Chinese Medicine (Beijing), Xiyuan Hospital, China Academy of Traditional Chinese Medicine (Beijing), China-Japan Friendship Hospital (Beijing), Beijing Hospital of Traditional Chinese Medicine, Capital University of Medical Sciences (Beijing), and the First Affiliated Hospital of Tianjin University of Traditional Chinese Medicine (Tianjin), the First Affiliated Hospital of Anhui University of Traditional Chinese Medicine (Anhui), the Affiliated Hospital of Liaoning University of Traditional Chinese Medicine (Liaoning), the Affiliated Hospital of Shandong University of Traditional Chinese Medicine (Shandong), and the Southwest Hospital of the Army Medical University (Chongqing).

The level of disease activity was determined using the ASDAS-CRP, with a score above 2.1 indicating high disease activity and below 2.1 indicating moderate or low disease activity. The cutoff value of 2.1 for ASDAS-CRP was selected based on the previously validated threshold defining high disease activity. The diagnostic criteria for AS followed the revised New York criteria (1984) and the classification criteria for axial spondyloarthritis recommended by the Assessment of ASAS (2009) [17, 18]. Inclusion criteria were: (1) meeting the diagnostic criteria for AS and (2) being at least 18 years of age. Exclusion criteria were: (1) having concurrent autoimmune diseases such as rheumatoid arthritis and (2) having incomplete medical records. This study was conducted after obtaining informed consent and written informed consent was obtained from all participants prior to enrollment., and it received ethical approval from the Ethics Committee of Guang’anmen Hospital, China Academy of Traditional Chinese Medicine (2022–108-KY). All methods were performed in accordance with the relevant guidelines and regulations.

### 2.2 Outcome and feature selection

We have defined two disease outcomes: (1) low activity or disease remission (ASDAS-CRP < 2.1) and (2) moderate or high disease activity (ASDAS-CRP ≥ 2.1). Our machine learning models considered a range of features, including both routinely recorded clinical data and PROMs. Clinicians regularly recorded demographic information (age, gender), basic patient details (height, body mass index, smoking status, family history, disease duration), comorbidities (hypertension, diabetes mellitus, hyperlipidemia, stroke), extra-articular manifestations (ophthalmia, intestinal infections, urinary infections), and key symptomatic inquiries (fatigue levels, low back pain, peripheral joint pain or swelling, degree and duration of morning stiffness). Eight PROMs were incorporated into the machine learning model for feature selection, including the Bath Ankylosing Spondylitis Disease Activity Index (BASDAI), BASFI, and Bath Ankylosing Spondylitis Metrology Index (BASMI). Additionally, patient-reported outcomes included the Patient Global Assessment (PGA), Nocturnal Pain Score (VAS), Ankylosing Spondylitis Health Index (ASAS-HI), and the Functional Assessment of Chronic Illness Therapy Fatigue Scale (FACIT-F). Psychological status was evaluated using the Depression Anxiety Stress Scales (DASS-21).

### 2.3 Data pre-processing and standardization

Following data collection, a comprehensive data cleaning and pre-processing procedure was carried out. The data cleaning process involved identifying and addressing any missing values, duplicates, and outliers. To minimize their impact on subsequent analyses, missing values were handled through deletion. Outliers were also detected and corrected to prevent their influence on model training and prediction accuracy. After the cleaning phase, the data was standardized or normalized to eliminate any differences in feature magnitudes. This allowed for more consistent comparisons and analyses across variables.

### 2.4 Model construction and model performance evaluation

**Fig 1:**
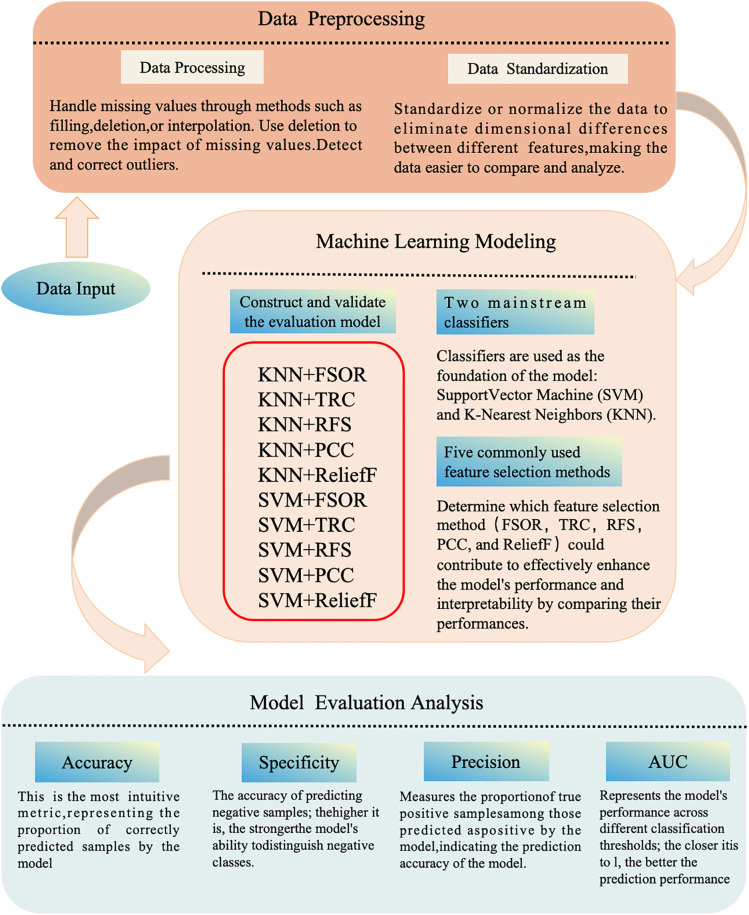
Model construction flowchart. Note: SVM: Support Vector Machine; KNN: K-Nearest Neighbour; FSOR: Feature Selection with Orthogonal Regression; TRC: Trace Ratio Criterion; RFS: Robust Feature Selection; PCC: Pearson Correlation Coefficient; AUC: area under the curve.

The model construction process is illustrated in Fig 1. The data collected from the enrolled patients was divided into a training set (n = 311) and a validation set (n = 78) using an 80:20 ratio. The model development process involved two primary components: feature selection and classification. Five widely-used widely used feature selection methods—Feature Selection with Orthogonal Regression (FSOR), Trace Ratio Criterion (TRC), Robust Feature Selection (RFS), Pearson Correlation Coefficient (PCC), and ReliefF—were employed, which represent filtering and embedding techniques, respectively [19–23]. These methods were applied to the dataset to identify and rank features based on their importance in the AS disease activity prediction task. The selected features were then inputted into two classifiers, the Support Vector Machine (SVM) and K-Nearest Neighbors (KNN), to evaluate and test their classification accuracy. Each model was responsible for directly classifying the ASDAS-CRP categories and then mapping the predicted ASDAS-CRP values to their respective categories. The performance of the models in the training set was evaluated by calculating the classification error rates to identify the most accurate model. The model’s performance was further validated in the validation set, where the model with the highest area under the receiver operating characteristic curve (AUC-ROC) was selected as the best-performing model. To ensure stability and reliability, each model was run 50 times using different random seeds and the average results were computed as the final outputs. In this study, the prediction models were evaluated using sensitivity, specificity, and accuracy metrics.

## 3 Results

### 3.1 Feature importance ranking

**Fig 2:**
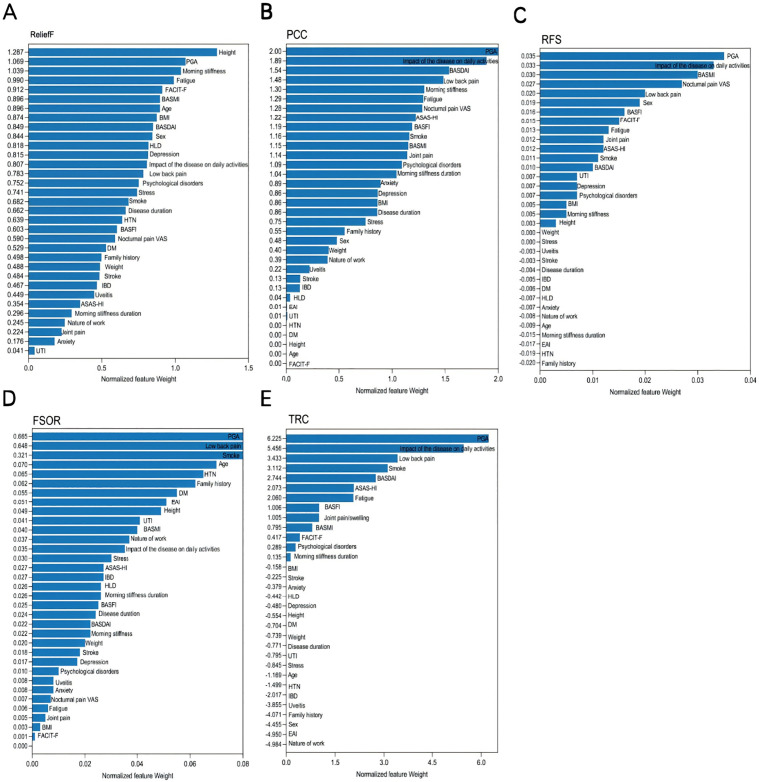
Average feature importance assessment for collective feature selection. ReliefF, B.PCC, C.RFS, D.FSOR, E.TRC. Note: The abscissa is the proportion score of evaluating the importance of features, and the ordinate is the top 34 features with the highest score.PCC: Pearson Correlation Coefficient; RFS: Robust Feature Selection; FSOR: Feature Selection with Orthogonal Regression; TRC: Trace Ratio Criterion.

We utilized five different feature selection methods (FSOR, TRC, RFS, PCC, and ReliefF) to determine the importance of each feature. Each feature with a score above zero was considered potentially relevant and is listed in Fig 2. ReliefF identified 34 variables, including Height, PGA, Morning Stiffness, Fatigue, FACIT-F, BASMI, Age, BMI, BASDAI, and Sex. PCC identified 29 variables, which included PGA, the impact of the disease on daily activities, BASDAI, Low Back Pain, Morning Stiffness, Fatigue, Nocturnal Pain VAS, ASAS-HI, BASFI, Smoking status, and BASMI. RFS identified 19 variables, comprising PGA, the impact of the disease on daily activities, BASMI, Nocturnal Pain VAS, Low Back Pain, Sex, BASFI, FACIT-F, Fatigue, and Joint Pain. FSOR identified 33 variables, including PGA, Smoking status, the impact of the disease on daily activities, BASMI, Nocturnal Pain VAS, Low Back Pain, Sex, BASFI, and FACIT-F. Finally, TRC identified 13 variables, which included PGA, the impact of the disease on daily activities, Low Back Pain, Smoking status, BASDAI, ASAS-HI, Fatigue, BASFI, Joint Pain, and BASMI.

### 3.2 Model performance validation

In this study, ten ML models were developed to predict high disease activity in AS. To evaluate the models in the test set, we employed a combination of dichotomous metrics and visual assessments to provide a comprehensive overview of the models, including accuracy, specificity, sensitivity, and AUC. Additionally, we visualized the ROC curves for all algorithms and evaluated model performance using AUC.

**Fig 3:**
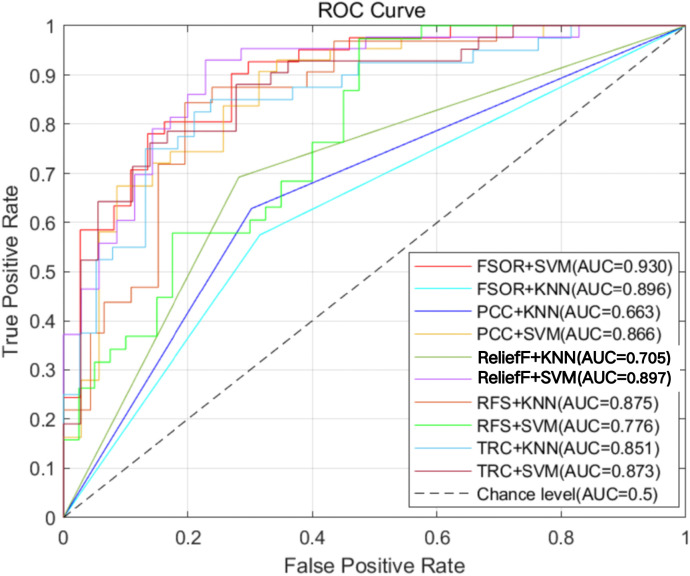
In the ROC plot, we present 10 models and use the area under the curve (AUC) to evaluate the performance of the models. Note: SVM: Support Vector Machine; KNN: K-Nearest Neighbour; FSOR: Feature Selection with Orthogonal Regression; TRC: Trace Ratio Criterion; RFS: Robust Feature Selection; PCC: Pearson Correlation Coefficient; AUC: area under the curve; ROC: Receiver Operating Characteristic.

Fig 3 illustrates the varying performance of all models in terms of ROC curves. Among these ten models, the FSOR+SVM (AUC = 0.930, 95%CI: 0.87–0.99) emerged as the most effective predictor of high disease activity in AS, followed by ReliefF + SVM (AUC = 0.897, 95%CI: 0.83–0.96), FSOR+KNN (AUC = 0.896, 95%CI: 0.83–0.96), RFS + SVM (AUC = 0.875, 95%CI: 0.80–0.95), TRC + SVM (AUC = 0.873, 95%CI: 0.80–0.95), PCC + SVM (AUC = 0.866, 95%CI: 0.79–0.94), TRC + KNN (AUC = 0.851, 95%CI: 0.77–0.93), RFS + KNN (AUC = 0.776, 95%CI: 0.68–0.87), ReliefF + KNN (AUC = 0.705, 95%CI: 0.60–0.81), and PCC + KNN (AUC = 0.663, 95%CI: 0.56–0.77). These models represent the optimal diagnostic performance in this study.

### 3.3 Other performance evaluation of the model

Two classifiers, SVM and KNN, were utilized in combination with five feature selection methods FSOR, TRC, RFS, PCC, and ReliefF to create ten combined models: KNN+FSOR, KNN + TRC, KNN + RFS, KNN + PCC, KNN+ReliefF, SVM+FSOR, SVM + TRC, SVM + RFS, SVM + PCC, and SVM+ReliefF. The accuracy of the five combinations involving SVM classifiers was significantly higher than that of the five combinations involving the KNN classifier (Table 1).

**Table 1:** The specificity, sensitivity and average accuracy of the models were measured（%）.

	Accuracy	Specificity	Sensitivity
PCC + SVM	70.74	75.61	53.28
PCC + KNN	74.48	60.94	82.18
ReliefF + SVM	77.38	80.31	62.61
ReliefF + KNN	74.01	83.19	35.68
RFS + SVM	76.33	78.95	82.50
RFS + KNN	71.17	65.00	73.68
TRC + SVM	78.15	88.10	75.00
TRC + KNN	69.53	71.79	53.85
FSOR+KNN	73.60	85.71	67.44
FSOR+SVM	**79.35**	**90.50**	**83.78**

Variations in parameter settings and internal algorithms contributed to the differing performance of each model. The specificity and sensitivity metrics for the ten models are presented in Table 1. The FSOR+SVM model exhibited superior sensitivity and specificity, indicating its greater stability. Among the models, the FSOR+SVM model demonstrated the highest sensitivity (83.78%), accuracy (79.35%), and specificity (90.50%), suggesting that it is the optimal model with substantial clinical utility.

## 4. Discussion

The ASDAS-CRP is commonly employed to assist clinicians in making treatment decisions and monitoring patients’ conditions [24]. However, it is not uncommon for patients to struggle with calculating the ASDAS-CRP due to the absence of laboratory tests in routine clinical practice. In this study, we have developed and validated a straightforward and practical algorithm for predicting disease activity in AS using a machine learning approach. This model was based on features from PROMs and physicians’ routine records. The results demonstrate that our machine learning model, utilizing PROMs and routine records, can accurately and reliably predict AS disease activity.

PROs have been widely adopted by clinicians as important indicators in the clinical management of AS [25]. PROMs have gained increasing attention in both clinical practice and clinical trials [26, 27]. These measures represent direct reports from patients regarding their health status, symptoms, and treatment outcomes and do not rely on physician interpretation or assessment [28]. This self-reported approach provides firsthand information on patients’ subjective experiences and reflects the true quality of life and symptom changes during the treatment process. The application of PROMs in disease management not only enhances patient engagement and satisfaction but also provides clinicians with a critical basis for decision-making [27]. In this study, we specifically emphasize the importance and necessity of using PROMs to measure AS disease activity. First, PROMs can comprehensively and, in real-time reflect patients’ health status and quality of life, providing richer and more detailed information than traditional laboratory tests. For AS -- especially those who are unable to attend frequent medical appointments or undergo laboratory tests, assessing disease activity through PROMs is particularly vital.

In this study, data from 389 patients with AS were analyzed, and multiple prediction models were constructed using two classifiers – SVM and KNN -in combination with five feature selection methods (FSOR, TRC, RFS, PCC, and ReliefF). The results from the test set showed that the SVM classifier combinations had high accuracy, with the FSOR+SVM model performing the best in both the training and validation cohorts. This model achieved an AUROC of 0.930, indicating that a judicious combination of feature selection methods and classifiers can greatly improve the predictive performance of the models.

The PROMs-based machine learning model offers several advantages over traditional methods of assessing disease activity in AS. First, the model does not rely on laboratory data; accurate predictions of disease activity can be achieved using only PROMs and routine clinical records. Second, the machine learning approach can process large datasets and capture complex feature relationships, resulting in more personalized and accurate predictions. This has important practical implications for patients who are unable to frequently visit healthcare providers or undergo laboratory tests for various reasons. The model not only alleviates patient burden by reducing reliance on frequent laboratory tests but also facilitates personalized disease management plans by capturing subjective patient perceptions and symptom changes. Furthermore, the ease of data collection associated with PROMs can significantly improve patient compliance and data availability, providing clinicians with a more reliable tool for assessing disease activity. Our approach is, therefore, highly feasible in scenarios where laboratory data may be unavailable or severely limited.

The results of this study indicate that a machine learning model based on PROMs and clinical records can be effectively used to predict disease activity in individuals with AS. This has several potential benefits. Firstly, healthcare professionals can utilize the model to promptly identify changes in patients’ conditions and adjust treatment plans accordingly, ultimately leading to improved patient outcomes. Secondly, the application of this model can reduce the need for frequent laboratory tests, resulting in lower medical costs and more efficient utilization of medical resources. Lastly, the inclusion of PROMs in the assessment method allows for a more comprehensive understanding of patients’ health status and quality of life, as it captures their subjective experiences.

The demand for telemedicine in the field of rheumatology is increasing, and the extension of this study’s findings is expected to improve the efficiency of remote patient monitoring [29, 30]. All variables used in the machine learning model can be obtained from rheumatologists’ electronic medical records, as well as easily and quickly from the patient-reported outcomes of rheumatology patients. The combination of routinely recorded clinician characteristics and PROMs data can effectively replace clinician-derived disease activity data through advanced analytics, accurately classifying AS disease activity and monitoring disease conditions while assessing treatment responses. Further validation using similar datasets in routine care settings may enhance the usefulness of this approach and support its practical application in remote patient monitoring programs or real-world evidence-based trials.

We acknowledge that our model requires completion of eight PROM instruments, which imposes greater time and cognitive burden on patients compared to using the single BASDAI. A legitimate question is whether the additional questionnaires provide sufficient incremental value to justify this burden. While a direct head-to-head comparison with BASDAI is beyond the scope of the current study, we note that our model’s AUC of 0.930 suggests potential added value from the multidimensional assessment. For clinical scenarios where detailed phenotyping is valuable (e.g., clinical trials, comprehensive baseline assessments), the additional burden may be justified. For routine telemedicine follow-up, a simplified BASDAI-only approach or a reduced PROM set may be more practical. Future studies should directly compare the two approaches head-to-head and explore model compression techniques to identify the minimal set of PROM items required for accurate prediction.

Some predictors in our model (e.g., BASFI, ASAS HI, HADS-depression) showed relatively lower feature importance weights in certain analyses. While this might suggest limited marginal contribution to predicting current ASDAS-CRP specifically, we retained them for two reasons. First, in ensemble machine learning methods, predictors with small but non-zero weights can still contribute to model calibration and stability—improvements that are not fully captured by AUC alone. Second, from a clinical perspective, these variables capture important dimensions of AS that extend beyond current disease activity: BASFI reflects functional decline from cumulative disease activity over time; ASAS HI captures the global impact of AS on health; and HADS-depression assesses mental health, which is increasingly recognized as integral to AS management. Removing them might simplify the model but could sacrifice clinically relevant information. Future studies could apply stricter feature selection (e.g., LASSO with higher penalty) to determine whether a smaller PROM set achieves comparable predictive performance without these dimensions.

This study has yielded valuable results; however, several limitations remain to be addressed. First, while we employed 5-fold cross-validation to mitigate overfitting, the relatively modest sample size (particularly the 78-case test set) may still affect the generalizability of our findings. Future studies should validate these models in larger, independent, multi-center cohorts. Second, the BASMI variable requires physical examination and is not a true patient-reported outcome measure, which limits the model’s applicability in pure telemedicine settings where only remote data collection is possible. Future research should develop PROM-only models by either excluding BASMI or identifying patient-reported surrogate measures for axial mobility (e.g., self-reported morning stiffness duration or patient-perceived flexibility). Third, our models lack external validation on an entirely independent dataset. External validation is essential before any clinical implementation, and we are currently planning such a study in collaboration with another rheumatology center. Fourth, the study sample was limited to clinical notes from Chinese rheumatologists, which may not accurately represent clinical practices in other countries. Therefore, future research should expand to include data from a more diverse range of countries for validation. Fifth, while the predictive performance of the model is already high, there is still potential for improvement. In subsequent studies, more advanced feature selection and classification algorithms should be explored to further optimize the model.

In summary, this study has successfully developed an efficient model for predicting disease activity in AS by integrating PROMs and clinical record features using a machine-learning approach. This model provides clinicians with a convenient and accurate tool for assessing patients’ disease activity, which can ultimately improve treatment outcomes and patient prognosis. However, further research and optimization are necessary to improve the model’s performance and applicability. Future studies could expand the sample size, optimize feature selection methods and model algorithms, and integrate biomarkers and other multi-source data to ensure its reliability and validity for a wider range of clinical applications.
